# Leveraging effect size distributions to improve polygenic risk scores derived from summary statistics of genome-wide association studies

**DOI:** 10.1371/journal.pcbi.1007565

**Published:** 2020-02-11

**Authors:** Shuang Song, Wei Jiang, Lin Hou, Hongyu Zhao

**Affiliations:** 1 Center for Statistical Science, Tsinghua University, Beijing, China; 2 Department of Industrial Engineering, Tsinghua University, Beijing, China; 3 Department of Biostatistics, School of Public Health, Yale University, New Haven, Connecticut, United States of America; University of Virginia, UNITED STATES

## Abstract

Genetic risk prediction is an important problem in human genetics, and accurate prediction can facilitate disease prevention and treatment. Calculating polygenic risk score (PRS) has become widely used due to its simplicity and effectiveness, where only summary statistics from genome-wide association studies are needed in the standard method. Recently, several methods have been proposed to improve standard PRS by utilizing external information, such as linkage disequilibrium and functional annotations. In this paper, we introduce EB-PRS, a novel method that leverages information for effect sizes across all the markers to improve prediction accuracy. Compared to most existing genetic risk prediction methods, our method does not need to tune parameters nor external information. Real data applications on six diseases, including asthma, breast cancer, celiac disease, Crohn’s disease, Parkinson’s disease and type 2 diabetes show that EB-PRS achieved 307.1%, 42.8%, 25.5%, 3.1%, 74.3% and 49.6% relative improvements in terms of predictive *r*^2^ over standard PRS method with optimally tuned parameters. Besides, compared to LDpred that makes use of LD information, EB-PRS also achieved 37.9%, 33.6%, 8.6%, 36.2%, 40.6% and 10.8% relative improvements. We note that our method is not the first method leveraging effect size distributions. Here we first justify our method by presenting theoretical optimal property over existing methods in this class of methods, and substantiate our theoretical result with extensive simulation results. The R-package EBPRS that implements our method is available on CRAN.

## Author summary

Genetic risk prediction is of considerable importance in human genetics. Calculating polygenic risk score (PRS) is the most commonly used approach due to its simplicity as well as the computational efficiency. Many PRS calculation methods have been proposed for accurate prediction by borrowing information from external panels or datasets. Here, instead of external information, we propose a novel PRS method leveraging internal information, namely the effect size distributions, to improve prediction accuracy. Neither parameter tuning nor external information is needed for our method. Extensive simulations and real data applications demonstrate that our method has substantial improvement over existing methods in terms of prediction accuracy.

## Introduction

The onset of common diseases results from the combined effects of genetic and environmental factors. The initial objective of genome-wide association studies (GWAS) is to identify genetic variants, such as single nucleotide polymorphisms (SNPs), that are associated with human diseases. In the past decade, GWAS have identified tens of thousands of genetic associations [1]. These identified associations have led to new insights on the etiologies of many diseases [2–4]. In addition to understanding the genetic basis of complex diseases, the association results provide us with an opportunity to develop genetic risk prediction models that are clinically useful. With accurate risk prediction methods, we can better advise individuals on appropriate prevention, screening, and diagnosis, and the estimated disease risk may also be helpful for treatment. For example, individuals with high predicted risk of cardiovascular disease or diabetes would be recommended for healthy diets, fitness regimens, as well as more regular check ups.

Calculating polygenic risk score (PRS) is a common genetic risk prediction approach because of its simplicity and computational efficiency. PRS sums the number of risk alleles among a set of SNPs, weighted by their effect sizes estimated from a certain cohort. We can regard PRS as the estimated genetic liability to a disorder or a trait [5]. The standard PRS method, namely the Pruning + Thresholding (P+T) method, selects SNPs after LD-clumping and *p*-value thresholding. The observed effect sizes of selected SNPs, which can be directly extracted from summary statistics, are used as weights. Hence, only GWAS summary statistics are needed to calculate PRS, which is attractive since summary statistics are more accessible than individual-level genotype data due to potential privacy and data sharing concerns. In the following, we only focus on the PRS methods utilizing GWAS summary statistics.

To further improve prediction accuracy, several methods have been proposed to utilize other information, such as LDpred (and LDpred-inf) that models the LD information extracted from a reference panel [6]; AnnoPred that leverages diverse types of genomic and epigenomic functional annotations [7]; and PleioPred and SMTpred that utilize pleiotropy relationship with other traits/diseases [8, 9]. All of these methods need to borrow information from external panels or datasets.

In this article, we explore whether we can improve the standard PRS method without using information from external panels or datasets. We give a positive answer to this question in this article by proposing EB-PRS, a novel PRS approach based on Empirical Bayes theory, borrowing information across markers to improve prediction accuracy. Instead of using a prespecified prior distribution in standard Bayesian modeling, the Empirical Bayes approach estimates the prior distribution from the data. Here we first utilize the GWAS summary statistics to infer the overall distribution of effect sizes and then leverage this distribution to improve the standard PRS method by minimizing the prediction error.

Compared to the existing genetic risk prediction methods, our method has the following advantages:

1. Our method does not need to tune parameters, avoiding the use of training data to select the best performing parameters.
2. Compared to other improved PRS methods such as LDpred and AnnoPred, we do not need external panels or datasets.
3. We note that we are not the first one proposing to utilize effect size distributions for PRS calculations. Some methods in this category also have no tuning parameters or external input, such as the methods proposed in So and Sham (2017) [10] and Mak et al. (2016) [11]. We theoretically construct the optimal PRS in terms of minimizing the prediction error, thus our method has theoretical superiority compared with the existing methods in this category. Better experimental performance also supports our theoretical results.

We demonstrate the better performance of our method through both simulations and real data applications to six complex diseases including asthma (AS), breast cancer (BC), celiac disease (CEL), Crohn’s disease (CD), Parkinson’s disease (PD), and type 2 diabetes (T2D).

## Results

### Simulation experiments

#### Simulation based on independent SNP assumption

We first performed simulations to investigate the performance of our method when the markers are independent. In these simulations, we fixed the number of independent SNPs to *m* = 10, 000 and the causal SNP proportion was set to 0.1. For each SNP, its allele frequency was simulated from a uniform distribution *U*(0.05, 0.95) and its effect size was drawn from a point-normal mixture distribution, i.e., *μ* ∼ 0.9*δ*_0_ + 0.1*N*(0, 0.001*N*_*e*_). We further set the prevalence of the disease *κ* to be 1%. To explore the relationship between the prediction performance of our method and the sample sizes of training datasets, we varied the sample size from 2,000 to 8,000. We first set the control-to-case ratios (CCRs) to 1 in the training datasets. In order to simulate under a setting consistent with real data, we also performed simulations with lager CCRs (ranging from 2 to 4).

**Fig 1:**
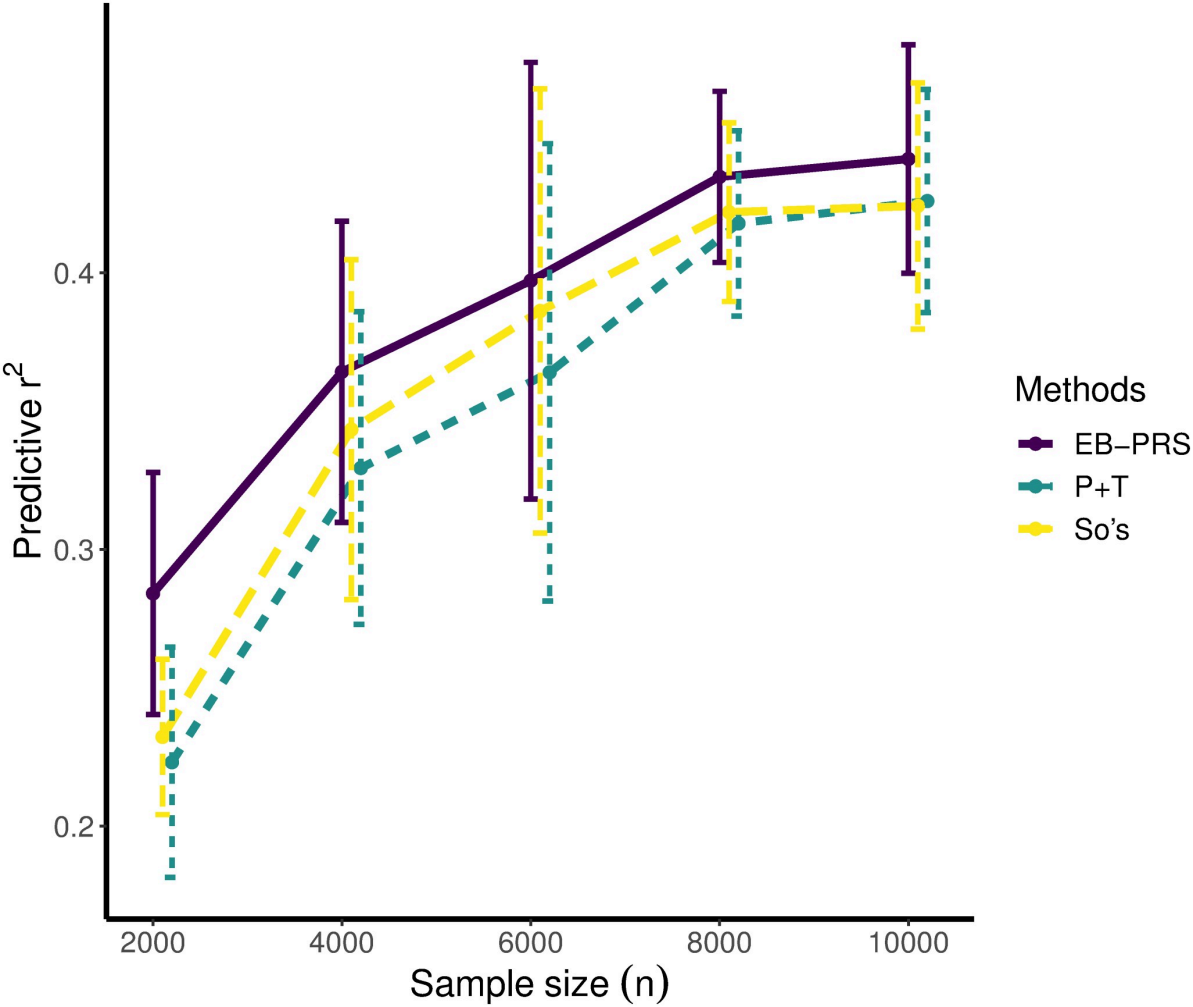
The average predictive r^2^ of the EB-PRS, P+T and So et al.’s method under different training sample sizes in simulation experiments with independent SNPs. Here the control-to-case ratio is set to one. EB-PRS always outperformed the other methods. The error bar indicates the standard deviation of predictive *r*^2^ across 10 times simulations.

**Fig 2:**
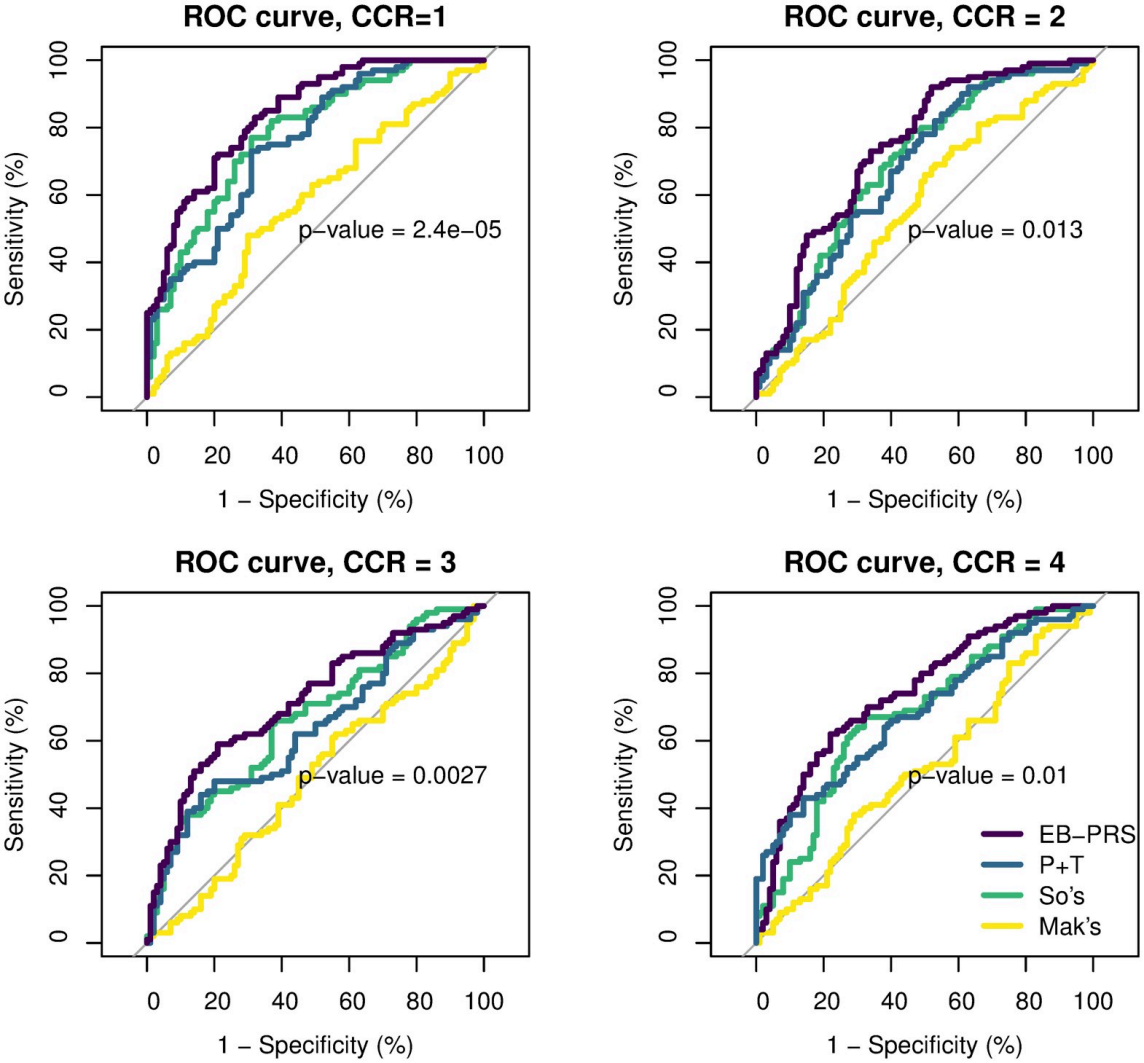
ROC curves of EB-PRS, P+T and methods from So et al. and Mak et al. under different CCRs in simulations with independent SNPs, when the training sample size is 2,000. We use the bootstrap-based method presented in Robin et al. [36] to compare the difference of AUC. We show the *p*-values of comparing the AUC of EB-PRS and P+T method.

Because SNPs were simulated independently, there is no need to consider LD among SNPs in this scenario. Therefore, we will only compare our method with a simplified P+T method in which the pruning step is not carried out. The *p*-value threshold of the P+T method was varied among {1, 5*e* − 01, 5*e* − 02, 5*e* − 03, 5*e* − 04, 5*e* − 05, 5*e* − 06}. We simulated 100 controls and 100 cases as our testing dataset. For each individual in the simulated testing dataset, the PRSs generated from the EB-PRS, P+T, So et al.’s and Mak et al.’s methods were calculated. We evaluate the prediction performance by using both the squared correlation between the PRSs and the observed phenotypes (predictive *r*^2^), and the area under the receiver operating characteristic (ROC) curve (AUC). For the P+T method, we report the results with the best performing parameters. We ran experiments in each setting 10 times and compared the average performances of four methods. Fig 1 shows the average predictive *r*^2^ of EB-PRS, P+T and the method from So et al. Results for using biobank-level sample sizes are in S1 Fig. We omit the results of Mak et al.’s method because it is far less competitive here. Fig 2 is the ROC curves under different CCRs when the sample size is 2,000. The average predictive *r*^2^ and AUCs for the four methods in different settings are summarized in S1 Table.

Population genetics data suggest that there are more SNPs with low minor allele frequencies (MAF) than those with high MAF [12]. To mimic this more realistic situation, we also simulate genotype data with allele frequencies from a scaled Beta distribution within (0.05, 0.95), where the density function is

$$\begin{array}{c} p\left(f\right)=\frac{1}{0.9B(a,b)}{\left(\frac{f-0.05}{0.9}\right)}^{a-1}{\left(\frac{0.95-f}{0.9}\right)}^{b-1}. \end{array}$$ (1)

Here we set shape parameters *a* = *b* = 0.8. The results of the simulation experiment are shown in S2 Fig. In addition, in real data, if the training and testing samples come from different populations, the allele frequencies of SNPs may be different, together with the causal variants and their effect sizes. Thus, we present the results when the distribution allele frequencies in the training set (uniform distribution) and the testing set (Beta distribution) are different, in S3 Fig. In addition, we specify the effect size of each causal SNP in the testing population different with their original value in the training population, and the difference between them follows a normal distribution *N*(0, 0.0005*N*_*e*_). The results showing the performance of different methods in divergent effect sizes can be seen in S4 Fig. We summarize the AUC and predictive *r*^2^ under different CCRs in Supplementary S2–S4 Tables. In addition, we present the performances of the four methods under different causal SNP proportions in Supplementary S5 Table. Under all circumstances of simulations, EB-PRS outperformed the other three methods.

#### Simulations based on real genotypes

In order to evaluate the performance of our method for depenent SNPs (i.e., SNPs are in LD), we conducted simulations based on individual-level genotype data accessed from the database of Genotypes and Phenotypes (dbGaP) [13, 14] (study accession number phs000021). This schizophrenia study dataset included 2,729 samples, and consisted of 729,454 SNPs. The CCR for the schizophrenia dataset is 1.2. We randomly selected 0.1% SNPs to have effects on disease and set the prior of the case proportion to 0.5. For these SNPs, their log-ORs (*β*) for associated SNPs were assumed to follow a normal distribution *N*(0, 0.04). The phenotype of each individual was generated according to the following formula:

$$\begin{array}{c} log\;(\frac{P(y=1|x)}{P(y=0|x)})=\sum_{i\in C}\beta_{i}x_{i}, \end{array}$$ (2)

where C is the set of causal SNPs. With this setting, the corresponding heritability in the observed scale is 49.2%.

Here we compare EB-PRS with six other methods including unadjusted PRS, P+T, LDpred-inf, LDpred, So et al.’s method, and Mak et al.’s method. We used genotype data of individuals with European ancestry from the 1000 Genomes Project as the reference panel for both LDpred-inf and LDpred. For LDpred, we set the proportion of causal SNPs from {1, 3*e* − 01, 1*e* − 01, 3*e* − 02, 1*e* − 02, 3*e* − 03, 1*e* − 03, 3*e* − 04, 1*e* − 04, 3*e* − 05, 1*e* − 05}. The five-fold cross validation was used to evaluate the prediction performance of different methods. For each training dataset, we calculated the summary statistics from the genotype data and utilized them to derive PRSs. The performance is measured using both the predictive *r*^2^ and AUC. For the P+T and LDpred, we report the results with the best performing parameters.

**Fig 3:**
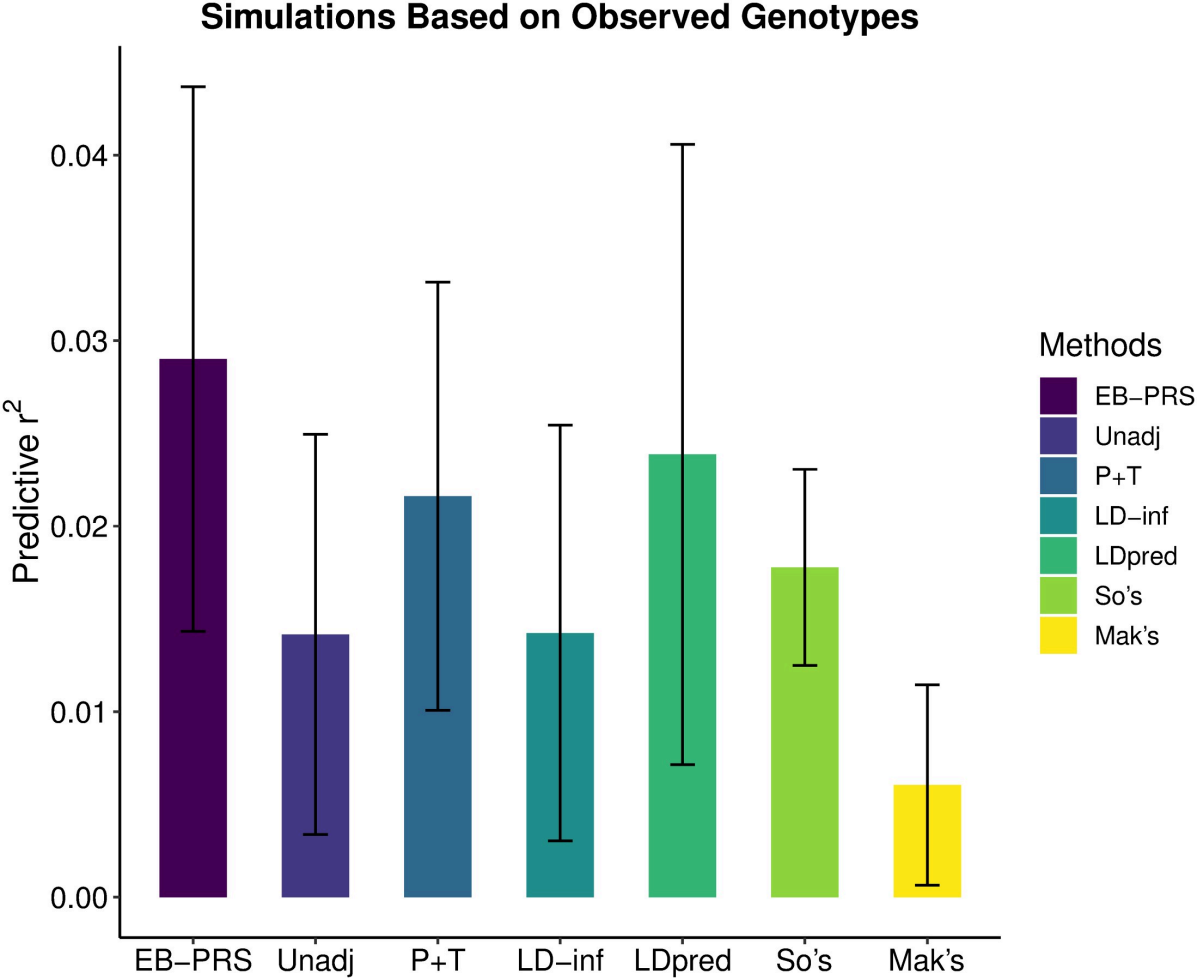
Predictive r^2^ of EB-PRS and six other methods on simulations based on observed genotypes using five-fold cross validation. The error bar indicates the standard deviation of predictive *r*^2^.

Fig 3 shows the predictive *r*^2^ of the seven methods using five-fold cross validation. We can see that EB-PRS is the best among the seven approaches. The exact values of predictive *r*^2^ and AUC are shown in Table 1. EB-PRS achieved 107%, 32%, 107%, 21%, 61%, and 383% relative improvements over the other six methods using the *r*^2^ metric. The AUC in the table also shows the predictive superiority of EB-PRS.

**Table 1:** Predictive r^2^ and AUC of EB-PRS, unadjusted PRS, P+T, LDpred-inf, LDpred So et al.’s method and Mak et al.’s method on simulations based on observed genotypes using five-fold cross validation. The simulations were based on individual-level genotype data accessed from the schizophrenia study (study accession number phs000021) in dbGaP. The dataset included 2,729 samples, and consisted of 729,454 SNPs. The highest mean *r*^2^ and AUCs are highlighted in boldface.

	EB-PRS	Uadj PRS	P+T	LDpred-inf	LDpred	So’s	Mak’s
Predictive *r*^2^	**0.029**	0.014	0.022	0.014	0.024	0.018	0.006
AUC	**0.633**	0.582	0.608	0.582	0.612	0.600	0.545

To help the user budget computation, we provide a summary table of computational time for our methods, LDpred and So et al.’s method for this simulation based on five-fold and ten-fold cross validation in Supplementary S6 Table. The simulations were based on an Intel Xeon processor with 2.50GHz.

### Applications to six real datasets

We applied EB-PRS and other PRS methods to six complex disease data sets including asthma (AS) [13], breast cancer (BC) [15, 16], celiac disease (CEL) [17], Crohn’s disease (CD) [18, 19], Parkinson’s disease (PD) [20] and type 2 diabetes (T2D) [21, 22] to illustrate the improved risk prediction performance in real data. We summarize the information of the training and testing sets in Table 2 and list details as below.

**Table 2:** Summary of the training summary statistics and the testing genotype data in real data applications.

Disease	Training	Sample Size	Number of SNPs	Testing	Sample Size
AS	GABRIEL Consortium	*n*_0_ = 16, 110 *n*_1_ = 10, 365	535, 060	dbGaP (phs000490)	*n*_0_ = 875 *n*_1_ = 515
BC	GAME-ON study	*n*_0_ = 41, 355 *n*_1_ = 16, 003	2, 435, 470	CGEMS	*n*_0_ = 70 *n*_1_ = 966
CEL	Dubois’ study	*n*_0_ = 10, 750 *n*_1_ = 4, 533	508, 742	NIDDK celiac disease study	*n*_0_ = 530 *n*_1_ = 1, 716
CD	IIBDGC (WTCCC removed)	*n*_0_ = 15, 056 *n*_1_ = 6, 333	871, 743	WTCCC	*n*_0_ = 2, 891 *n*_1_ = 1, 689
PD	Simon-Sanchez J et al.’s study	*n*_0_ = 3, 978 *n*_1_ = 1, 713	450, 439	WTCCC2	*n*_0_ = 2, 574 *n*_1_ = 1, 843
T2D	DIAGRAM	*n*_0_ = 56, 862 *n*_1_ = 12, 171	2, 400, 624	Northwestern NUgene Project	*n*_0_ = 517 *n*_1_ = 662

For AS, we trained the model using summary statistics from the GABRIEL Consortium, which was a meta-analysis of 23 studies, including 10,365 persons with physician-diagnosed asthma and 16,110 unaffected persons. There were 535,060 SNPs in the released summary statistics. We tested the performance utilizing samples from the study phs000490 in the dbGaP, with 515 cases and 875 controls [13]. For BC, we used summary statistics from the Genetic Associations and Mechanisms in Oncology (GAME-ON) study (*n*_0_ = 41, 335 and *n*_1_ = 16, 003) [15] covering 2,435,470 SNPs as the training data, and samples from the Cancer Genetic Markers of Susceptibility (CGEMS) study (*n*_0_ = 70, *n*_1_ = 966) [16] as the testing data. Shared individuals between two studies were removed in the testing data. For CEL, we trained the model with 4,533 individuals with celiac disease and 10,750 control from Dubois’ study [17], in which 508,742 SNPs were genotyped. The testing data is from samples in the National Institute of Diabetes and Digestive and Kidney Diseases (NIDDK) celiac disease study (1,716 cases and 530 controls) [23]. For CD, we trained the model using summary statistics from the International Inflammatory Bowel Disease Genetics Consortium (IIBDGC; *n*_0_ = 15, 056, *n*_1_ = 6, 333 and *m* = 871, 743) [18]. Individuals from the WTCCC were removed from the meta-analysis and used as the testing dataset (*n*_0_ = 2, 891, *n*_1_ = 1, 689) [19]. For PD, we used the summary statistics released from the Simon-Sanchez J et al.’s study as the training data, which summarized the association information of 450,439 SNPs genotyped among 1,713 individuals of European ancestry with PD and 3,978 controls with the same ancestry [20]. We used the Wellcome Trust Case Control Consortium 2 (WTCCC2) data (*n*_0_ = 2, 574 and *n*_1_ = 1, 843) as the testing data. For T2D, we trained the model on summary statistics from the Diabetes Genetics Replication and Meta-analysis consortium (DIAGRAM, *n*_0_ = 56, 862, *n*_1_ = 12, 171) [21] and tested the model on samples from the Northwestern NUgene Project (*n*_0_ = 517, *n*_1_ = 662) [22]. There were 2,400,624 SNPs in the training data.

For these six diseases, EB-PRS outperformed all other methods, including the unadjusted PRS, P+T, LDpred-inf, LDpred, So et al.’s and Mak et al.’s methods. Even without external information or datasets, the performance of our method was better than best performing LDpred for all diseases. Here, we build two logistic models, one fits PRSs derived from one method only, while the other one fits PRSs derived from two methods. Then we use the likelihood ratio test to compare the difference of the prediction accuracy and report the corresponding *p*-value. EB-PRS attained significant improvement in prediction accuracy over P+T for AS (*p*-value = 4.5*e* − 03), BC (*p*-value = 1.1*e* − 02), CEL (*p*-value < 2.0*e* − 16), CD (*p*-value = 1.5*e* − 07), PD (*p*-value = 1.8*e* − 02), and T2D (*p*-value = 4.4*e* − 06).

**Fig 4:**
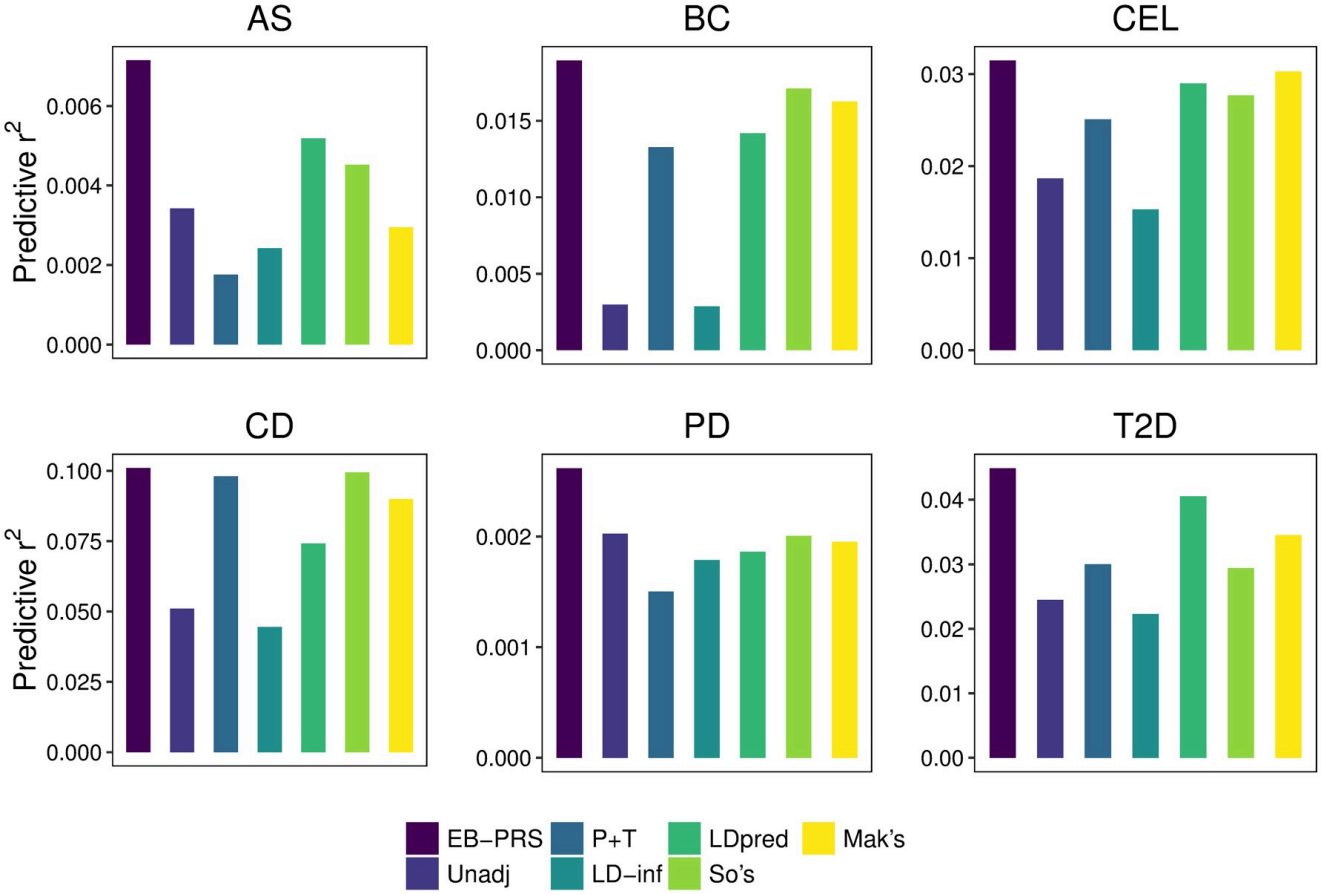
Comparisons of predictve r^2^ between EB-PRS and six other methods PRS on real data from six diseases.

Fig 4 shows the predictive *r*^2^ of all seven methods to six diseases. Each plot corresponds to a specific disease and the left column indicates the predictive accuracy of EB-PRS. We can see that EB-PRS always outperformed the other six methods. Specifically, evaluated by predictive *r*^2^, EB-PRS respectively achieved 307.1% (for AS), 42.8% (for BC), 25.5% (for CEL), 3.1% (for CD), 74.3% (for PD), and 49.6% (for T2D) relative improvements over P+T. Meanwhile, EB-PRS had 37.9% (for AS), 33.6% (for BC), 8.6% (for CEL), 36.2% (for CD), 40.6% (for PD), and 10.8% (for T2D) relatively improvement than LDpred. We also show the AUC values in Table 3. From the table we can see the superiority of EB-PRS over the other six methods.

**Table 3:** AUCs of different methods on real datasets of six diseases. The highest AUCs are highlighted in boldface.

Disease	EB-PRS	Uadj PRS	P+T	LDpred-inf	LDpred	So’s	Mak’s
AS	**0.550**	0.532	0.526	0.539	0.541	0.546	0.543
BC	**0.650**	0.551	0.629	0.551	0.628	0.640	0.640
CD	**0.687**	0.632	0.684	0.623	0.661	0.685	0.676
CEL	**0.621**	0.593	0.607	0.585	0.611	0.615	0.618
PD	**0.527**	0.520	0.525	0.518	0.519	0.521	0.522
T2D	**0.621**	0.586	0.595	0.581	0.614	0.594	0.604

In general, unadjusted PRS and LDpred-inf (without parameter tuning) performed poorly in all six datasets. Compared to methods which need to tune parameters (i.e., P+T and LDpred), our method also achieved better performance in all six datasets. These indicate the distinct advantage of our method: it can achieve noteworthy performance without tuning any parameters or incorporating external information.

## Discussion

In this article, we have considered predicting an individual’s genetic risk for complex diseases using summary statistics, which may prove helpful for both disease prevention and clinical decision. The PRS approach is widely used in genetic risk prediction because of its simplicity and efficiency, and a number of PRS methods have been proposed to improve prediction accuracy. Almost all these improved methods are based on borrowing information from external panels or datasets, such as the LD information, annotations or other GWAS datasets studying genetically correlated traits. In this paper we have proposed an improved PRS method by utilizing effect size distributions based on Empirical Bayes theory. No parameter tuning is needed, and no external panels or datasets are needed to be input. We have shown that our method outperformed the standard P+T method, as well as recently proposed methods in So et al. (2016) and Mak et al. (2017) in both simulations and real data analysis. Furthermore, compared to methods utilizing LD information from a reference panel, our method also achieved superior performance on all diseases including AS, BC, CEL, CD, PD, and T2D. One reason for the good performance is that we leverage the global information of effect sizes and the other reason is that we theoretically construct the optimal PRS in terms of minimizing the prediction error.

Note that both So et al.’s and Mak et al.’s methods also use effect size distribution to improve PRS. We show the theoretical superiority of our method over these two methods. So et al. used the square root of the explained liability as the effect size, which is not the optimal one in terms of minimizing prediction error. Besides, the effect size estimated from Tweedie’s formula has already been weighted by local true discovery rates, a further multiplication can make the effect sized over-shrinked. Meanwhile, their method needs to specify the prevalence for each trait, which may be difficult sometimes.

Some may argue that why we use parametric mixture model instead of non-parametric Tweedie’s formula in effect size estimation. First, in our model, we use symmetrical prior for the effect size, which makes the estimated magnitude invariant with the sign of ***z***, i.e., the selection of reference alleles. Second, the *K*-component mixture assumption for effect size of associated SNPs is consistent with the empirical observations from GWAS of many common diseases [24–26]. Third, although the Gaussian mixture model is parametric, it provides great flexibility and precision in modelling the underlying data. We can use this distribution to approximate arbitrary distribution to any fidelity with an appropriate number of mixtures [27].

Although our method can achieve better performance without tuning any parameters and utilizing external information, its performance may be improved with external information, for example, the LD information. Also, in order to increase the prediction accuracy to a larger extent, we may improve our method by combining other available datasets in the future, such as annotations or other GWAS summary statistics studying genetically correlated traits.

A basic assumption underlying all PRS methods is that the training and testing datasets are homogeneous and sampled from the same population. If they come from different populations, the allele frequencies of SNPs will be different, together with the causal variants and their effect sizes. In the case, we expect a worse prediction performance [28, 29]. In this article, we use simulation experiments to mimic the divergent situation between training and testing datasets. Different distributions of allele frequencies, as well as a divergency in effect sizes are simulated. Results summarized in S3 and S4 Tables showed that, although the prediction performance will be influenced due to the divergence of two sets, our EB-PRS method still outperforms the others. However, our current analysis still lacks the testing across diverse populations.

## Materials and methods

### EB-PRS

Generally speaking, EB-PRS aims at minimizing the prediction error by leveraging estimated distribution of effect sizes. Assuming that the SNPs are independent, we can show that the optimal PRS (in terms of achieving the best classification accuracy) is

$$\begin{array}{c} S=\beta^{T}x=\sum_{i=1}^{m}\beta_{i}x_{i}, \end{array}$$ (3)

where *m* is the total number of genotyped SNPs, *x*_*i*_ is the genotypic value, and *β*_*i*_ is the log-odds ratio (OR) of the *i*-th SNP. The log-OR is a measure of the effect size defined in the following formula:

$$\begin{array}{c} \beta_{i}=\text{log}\;(\frac{f_{i1}\left(1-f_{i0}\right)}{f_{i0}\left(1-f_{i1}\right)}), \end{array}$$ (4)

where *f*_*i*0_ and *f*_*i*1_ are the reference allele frequencies among controls and cases, respectively. If the SNP is not associated with disease, then *β*_*i*_ = 0.

In practice, the true values of effect sizes are usually unknown, and need to be estimated from the data. In our method, we use the Empirical Bayes approach to estimate ***β***, which is the minimizer of the Bayes risk under the distribution estimated from the data. The estimators can be directly derived from GWAS summary statistics.

#### Notations and assumptions

Suppose that there are *m* SNPs genotyped in a GWAS. For each SNP, there are usually two different alleles. We denote one of them as the reference allele, and use the number of reference alleles to code the genotype of the SNP for each individual. For SNP *i* (*i* = 1, …, *m*), the genotypic value is denoted by *x*_*i*_ (*x*_*i*_ ∈ {0, 1, 2}). We use ***x*** to represent the vector of genotypic values across *m* SNPs of an individual and *y* the disease status of the individual, where *y* = 1 if the individual has the disease and *y* = 0 otherwise.

With a multiplicative model and low prevalence, the genotypic value of each SNP follows binomial distribution as:

$$\begin{array}{c} X_{i}|\left(y=0\right)∼Bin\left(2,f_{i0}\right),\;X_{i}|\left(y=1\right)∼Bin\left(2,f_{i1}\right). \end{array}$$ (5)

#### Optimal polygenic risk score

If all SNPs are assumed to be independent, based on Eq (5), we have:

$$\begin{array}{c} \begin{array}{cc} \text{log}\;(\frac{P(y=1|x)}{P(y=0|x)}) & =\text{log}\;(\frac{\kappa }{1-\kappa })+\sum_{i=1}^{m}\text{log}\;(\frac{f_{i1}^{x_{i}}{\left(1-f_{i1}\right)}^{2-x_{i}}}{f_{i0}^{x_{i}}{\left(1-f_{i0}\right)}^{2-x_{i}}})\\ & =\text{log}\;(\frac{\kappa }{1-\kappa })+2\sum_{i=1}^{m}\text{log}\;(\frac{1-f_{i1}}{1-f_{i0}})+\sum_{i=1}^{m}x_{i}\beta_{i}, \end{array} \end{array}$$ (6)

where *κ* is the disease prevalence, and *β*_*i*_ is the true value of the log-OR for the *i*-th SNP.

Our objective is to find the decision rule minimizing the overall Bayes risk:

$$\begin{array}{c} R=\int R(\alpha (x)|x)p(x)dx. \end{array}$$ (7)

By Bayes decision rule, we minimize the overall risk by select the action that minimizes the conditional risk *R*(*α*(***x***)|***x***) for all ***x***:

$$\begin{array}{c} \alpha^{*}=\underset{\alpha_{l}}{\text{arg}\;\text{min}}\;R\left(\alpha_{l}|x\right)=\underset{\alpha_{l}}{\text{arg}\;\text{min}}\;[\lambda \left(\alpha_{l}|y=0\right)P\left(y=0|x\right)+\lambda \left(\alpha_{l}|y=1\right)P\left(y=1|x\right)], \end{array}$$ (8)

where *l* ∈ {0, 1} and *α*_0_ when $\hat{y}=0$ and *α*_1_ when $\hat{y}=1$. λ(⋅) is the Zero-One Loss Function:

$$\begin{array}{c} \lambda \left(\alpha_{l}|y=w\right)=\{\begin{array}{cc} 0 & l=w\\ 1 & l\neq w \end{array}. \end{array}$$ (9)

Thus, Eq (7) can be minimized by setting $\hat{y}=1$ if *P*(*y* = 1|***x***) > *P*(*y* = 0|***x***), i.e.,

$$\begin{array}{c} \text{log}\;(\frac{P(y=1|x)}{P(y=0|x)})=\text{log}\left(\frac{\kappa }{1-\kappa }\right)+2\sum_{i=1}^{m}\text{log}\;(\frac{1-f_{i1}}{1-f_{i0}})+\beta^{T}x>0, \end{array}$$ (10)

where ***β*** is exactly the log-OR. That is, if we define a polygenic risk score $S=\beta^{T}x=\sum_{i=1}^{m}\beta_{i}x_{i}$, the optimal decision rule minimizing the prediction error is

$$\begin{array}{c} \hat{y}=\{\begin{array}{cc} 1 & \text{if}\;S>-\text{log}\;(\frac{\kappa }{1-\kappa })-2\sum_{i=1}^{m}\text{log}\;(\frac{1-f_{i1}}{1-f_{i0}})\\ 0 & \text{otherwise}\; \end{array}. \end{array}$$ (11)

#### Inference

The constructed optimal polygenic risk score *S* is a function of the parameters *β*_*i*_ (*i* = 1, …, *m*). To derive the PRS in practice, we can use GWAS summary statistics to estimate these parameters.

If we simply estimate *β*_*i*_ by plugging the observed log-OR, the estimated effect sizes will tend to be inflated for SNPs with large values of estimated results. This phenomenon is commonly known as the “winner’s curse”. Here, we adopt the Empirical Bayes approach to address the issue of the selection bias as it is more robust to the winner’s curse [30, 31]. Also, it is the minimizer of the Bayes risk under the effect size distribution estimated from the data.

In GWAS, we usually use the following log-OR test to assess associations between SNPs and disease:

$$\begin{array}{c} z_{i}=\frac{\hat{\beta_{i}}}{se(\hat{\beta_{i}})}, \end{array}$$ (12)

where $se\left(\hat{\beta_{i}}\right)=\sqrt{\frac{2}{N_{e}f_{i}\left(1-f_{i}\right)}}$ and $N_{e}=\frac{4n_{0}n_{1}}{n}$ is the effective sample size in the case-control study, where *n*_0_ and *n*_1_ are the number of control and disease subjects, respectively, and *n* = *n*_0_ + *n*_1_. Given the standardized effect size $\mu_{i}=\frac{\beta_{i}}{se(\hat{\beta_{i}})}$, the distribution of *z*-scores is *Z*_*i*_|*μ*_*i*_ ∼ *N*(*μ*_*i*_, 1). Recent GWAS results suggest that, among all disease-associated SNPs, there are many more SNPs with small effect sizes than those with large effect sizes [32, 33]. Therefore, we use the following spike-and-slab prior to model the effect sizes of all SNPs:

$$\begin{array}{c} \mu_{i}∼\pi_{0}\delta_{0}+\sum_{j=1}^{K}\pi_{j}N\left(0,N_{e}\sigma_{j}^{2}\right), \end{array}$$ (13)

where *π*_0_ is the proportion of non-associated SNPs (0 ≤ *π*_0_ ≤ 1) and *δ*_0_ is the distribution with point mass at zero. Here we use a *K*-component Gaussian mixture distribution as the slab, in which the proportion of SNPs in the *j*-th associated component is *π*_*j*_ ($\sum_{j=0}^{K}\pi_{j}=1$) and the corresponding variance of the standardized effect sizes is $N_{e}\sigma_{j}^{2}$. We add *N*_*e*_ as a scaling coefficient in the variance of each component in order to make $\sigma_{j}^{2}$ invariant with changing sample size.

With this prior specification, the posterior expected effect size of an SNP under each alternative hypothesis is

$$\begin{array}{c} E\left(\beta_{i}|z_{i},H_{1j}\right)=\frac{N_{e}\sigma_{j}^{2}}{1+N_{e}\sigma_{j}^{2}}\hat{\beta_{i}}, \end{array}$$ (14)

where $H_{1j}:\mu_{i}\neq 0$ is the alternative hypothesis that the SNP is an associated one within the *j*-th component (*j* = 1, …, *K*). The corresponding local true discovery rate is the probability that the hypothesis $H_{1j}$ is true, given its *z*-value, and it can be calculated as follows:

$$\begin{array}{c} {\text{ltdr}}_{ij}=P\left(H_{1j}|z_{i}\right)=\frac{\pi_{j}f_{j}\left(z_{i}\right)}{\pi_{0}\phi \left(z_{i}\right)+\sum_{j=1}^{K}\pi_{j}f_{j}\left(z_{i}\right)}, \end{array}$$ (15)

where *ϕ*(⋅) and *f*_*j*_(⋅) are the probablity density functions of *N*(0, 1) and $N(0,1+N_{e}\sigma_{j}^{2})$, respectively.

The posterior expectation of *β*_*i*_ is the optimal estimator for minimizing the Bayes risk. Therefore, we estimate *β*_*i*_ as follows:

$$\begin{array}{c} E\left(\beta_{i}|z_{i}\right)=\sum_{j=1}^{K}{\text{ltdr}}_{ij}\cdot E\left(\beta_{i}|z_{i},H_{1j}\right). \end{array}$$ (16)

Here we adopt an EM-algorithm to estimate unknown parameters (*π*_0_, *π*_1_, ⋯, *π*_*K*_) and (*σ*_1_, ⋯, *σ*_*K*_) in the above mixture model. In practice, the null proportion *π*_0_ is always much larger than the proportions in the alternative components. To take advantage of this prior information, we first add a Dirichlet prior (*α*, 0, ⋯, 0) to proportions (*π*_0_, *π*_1_, …, *π*_*K*_). We use the following strategy to infer the value of *α*. First, we set *α* to a reasonable value ($\frac{m}{20}$ in our default setting) to obtain a preliminary model. Then we generate parametric bootstrap samples based on the preliminary model and select *α* with the minimal relative errors in terms of parameter estimation using the bootstrap samples [34].

Theoretically, a better fitting for the underlying distribution can be obtained by increasing the component number *K*. That can further improve the prediction performance. However, the model and computational complexity will also be increased accordingly. If we keep increasing *K* to a certain level, an original component will be divided into multiple similar components in the estimation, which does not add benefit to the prediction. In practice, we found that we achieved both the discriminability for components and the prediction performance when *K* = 3. Therefore, we set *K* to three as the default setting in our method.

Next, we compare our method named EB-PRS with six other methods: unadjusted PRS, P+T, LDpred-inf, LDpred, and two methods proposed in So et al. (2017) [10] and Mak et al. (2016) [11], respectively. In the following, we briefly describe these methods. We note that the last two methods were also proposed to utilize effect size distributions for PRS calculations, where no tuning parameters or external input is needed. We will discuss their differences with our method later.

### Unadjusted PRS

The unadjusted PRS is the summation of genotypic values weighted by the corresponding estimated effect sizes from the marginal logistic regression, i.e.,

$$\begin{array}{c} S_{unadj}={\hat{\beta }}^{T}x=\sum_{i=1}^{m}{\hat{\beta }}_{i}x_{i}, \end{array}$$ (17)

where ${\hat{\beta }}_{i}$ is the estimated coefficient from the marginal logistic regression for SNP *i*. If no confounders are considered in the regression, then ${\hat{\beta }}_{i}$ is equal to the log-OR.

### P+T

The P+T method generates PRS based on a subset of independent SNPs obtained via informed LD-clumping [35] and *p*-value thresholding. The method first prunes SNPs according to a given threshold for squared correlation coefficients (*r*^2^) between different SNPs. Then it further filters the SNPs with a *p*-value cutoff in marginal association tests. In this article, we set LD clumping *r*^2^ to 0.1 and vary the *p*-value cutoff values from {1, 5*e* − 01, 5*e* − 02, 5*e* − 03, 5*e* − 04, 5*e* − 05, 5*e* − 06}. In the following comparison experiments, we will report the prediction results of the P+T method with the optimally tuned parameters.

### LDpred-inf

The LDpred-inf method estimates the causal effect sizes under an infinitesimal model, where LD among SNPs is considered. The assumption for the causal effect size is $\beta_{i}∼N\left(0,\frac{h_{g}^{2}}{m}\right)$, where $h_{g}^{2}$ denotes the heritability explained by these SNPs. The posterior means of the causal effect sizes are

$$\begin{array}{c} E\left(\beta |\hat{\beta },D\right)={\left(\frac{m}{nh_{g}^{2}}I+D\right)}^{-1}\hat{\beta }. \end{array}$$ (18)

Here ***D*** denotes the LD matrix, *n* is the sample size of the GWAS, ***I*** denotes the identity matrix, and $\hat{\beta }$ is the vector of estimated effect sizes from marginal regression. The PRS is

$$\begin{array}{c} S_{LDpred-inf}=E{\left(\beta |\hat{\beta },D\right)}^{T}w. \end{array}$$ (19)

Here, ***w*** represents the corresponding standardized genotype.

### LDpred

LDpred is an extension of LDpred-inf, which makes use of a point-normal prior instead of normal prior to estimate causal effect sizes. To calculate the posterior expectation of causal effects, Markov Chain Monte Carlo (MCMC) is used, and the proportion of causal effects *π*_0_ is a parameter that needs to be specified by users. Here, we set *π*_0_ from {1, 3*e* − 01, 1*e* − 01, 3*e* − 02, 1*e* − 02, 3*e* − 03, 1*e* − 03, 3*e* − 04, 1*e* − 04, 3*e* − 05, 1*e* − 05}, which are the default values recommended in LDpred. In the following comparison experiments, we will report the best prediction results with the optimally tuned parameters.

### So et al.’s method

So et al. proposed to construct PRS by using the following three steps to calculate the effect size of each SNP. Firstly, the standardized effect size *μ* is non-parametrically estimated by using the Tweedie’s formula:

$$\begin{array}{c} E\left(\mu |z\right)=z+\frac{f^{\prime }\left(z\right)}{f(z)}, \end{array}$$ (20)

where *f*(*z*) is the estimated probability density function of *z*-values. Then the explained liability of each SNP is assessed based on estimated *μ*. The final effect size is estimated using the square root of explained liability weighted by the corresponding local true discovery rate, i.e.,

$$\begin{array}{c} {\tilde{\beta }}_{So}=\text{ltdr}\cdot \sqrt{\epsilon (E(\mu |z)}. \end{array}$$ (21)

Since we have theoretically shown that the true log-OR is the optimal effect size for constructing PRS in terms of prediction accuracy, using the square root of explained liability will decrease the performance. In addition, Tweedie’s formula evaluates the posterior expecation of *μ* without conditioning alternative hypothesis $H_{1}$, and we have

$$\begin{array}{c} E\left(\mu |z\right)=\text{ltdr}\cdot E\left(\mu |z,H_{1}\right). \end{array}$$ (22)

The further weighting strategy based on ltdr will make the effect size estimate over-shrinked.

### Mak et al.’s method

Mak et al. proposed to construct PRS by directly using the observed effect size weighted by ltdr, i.e.,

$$\begin{array}{c} {\tilde{\beta }}_{Mak}=\text{ltdr}\cdot \hat{\beta }. \end{array}$$ (23)

As we pointed out before, the observed effect size suffers from the winner’ curse. The shrinkage estimator derived from Empirical Bayes theory is a better choice in terms of overcoming the winner’s curse and minimizing the Bayes risk.

## Supporting information

S1 Table Predictive *r*^2^ and AUC of EB-PRS, P+T, So et al.’s method and Mak et al.’s method under different training sample sizes and control-to-case ratios (CCRs) in simulation experiments with independent SNPs. The allele frequencies follow uniform distribution. The highest mean *r*^2^ and AUCs are highlighted in boldface.
(XLSX)

S2 Table Predictive *r*^2^ and AUC of EB-PRS, P+T, So et al.’s method and Mak et al.’s method under different training sample sizes and control-to-case ratios (CCRs) in simulation experiments with independent SNPs. The allele frequencies follow Beta distribution. The highest mean *r*^2^ and AUCs are highlighted in boldface.
(XLSX)

S3 Table Predictive *r*^2^ and AUC of EB-PRS, P+T, So et al.’s method and Mak et al.’s method under different training sample sizes and control-to-case ratios (CCRs) in simulation experiments with independent SNPs. The allele frequencies of training data (uniform distribution) and testing data (Beta distribution) are different. The highest mean *r*^2^ and AUCs are highlighted in boldface.
(XLSX)

S4 Table Predictive *r*^2^ and AUC of EB-PRS, P+T, So et al.’s method and Mak et al.’s method under different training sample sizes and control-to-case ratios (CCRs) in simulation experiments with independent SNPs. Effect size differences among the populations were added among training and testing data. The allele frequencies follow uniform distribution in the training and testing data. The highest mean *r*^2^ and AUCs are highlighted in boldface.
(XLSX)

S5 Table Predictive *r*^2^ and AUC of EB-PRS, P+T, So et al.’s method and Mak et al.’s method under different different causal SNP proportions when CCR = 1 in simulation experiments with independent SNPs. The allele frequencies follow uniform distribution and the sample size here is 5,000. The results are the average of 10 times simulations. The highest mean *r*^2^ and AUCs are highlighted in boldface.
(XLSX)

S6 Table Computational time for EB-PRS, LDpred and So et al.’s method for this simulation based on five-fold and ten-fold cross validation in supplementary table. The simulations were based on an Intel Xeon processor with 2.50GHz. The shortest time is highlighted in boldface.
(XLSX)

S1 Fig The average predictive r^2^ of the EB-PRS, P+T and So et al.’s method using biobank-level sample sizes in simulation experiments with independent SNPs. Here the control-to-case ratio is set to one. EB-PRS always outperformed the other methods. So et al.’s package will produce errors when the sample size is above 3*e* + 05. The error bar indicates the standard deviation of predictive *r*^2^ across 10 times simulations.
(TIF)

S2 Fig The average predictive r^2^ of the EB-PRS, P+T and So et al.’s method under different training sample sizes in simulation experiments with independent SNPs. The allele frequencies in training and testing data follow Beta distribution. Here the control-to-case ratio is set to one. EB-PRS always outperformed the other methods. The error bar indicates the standard deviation of predictive *r*^2^ across 10 times simulations.
(TIF)

S3 Fig The average predictive r^2^ of the EB-PRS, P+T and So et al.’s method under different training sample sizes in simulation experiments with independent SNPs. The allele frequencies in training (uniform distribution) and testing (Beta distribution) data are different. Here the control-to-case ratio is set to one. EB-PRS always outperformed the other methods. The error bar indicates the standard deviation of predictive *r*^2^ across 10 times simulations.
(TIF)

S4 Fig The average predictive r^2^ of the EB-PRS, P+T and So et al.’s method under different training sample sizes in simulation experiments with independent SNPs. The effect size of each causal SNP in the training and testing populations is different. Here the control-to-case ratio is set to one. EB-PRS always outperformed the other methods. The error bar indicates the standard deviation of predictive *r*^2^ across 10 times simulations.
(TIF)
