# Supplementary figures and images for: Leveraging effect size distributions to improve polygenic risk scores derived from summary statistics of genome-wide association studies
Source: PLoS Comput Biol. 2020 Feb 11;16(2):e1007565. doi: 10.1371/journal.pcbi.1007565 (PMC7039528; doi:10.1371/journal.pcbi.1007565)

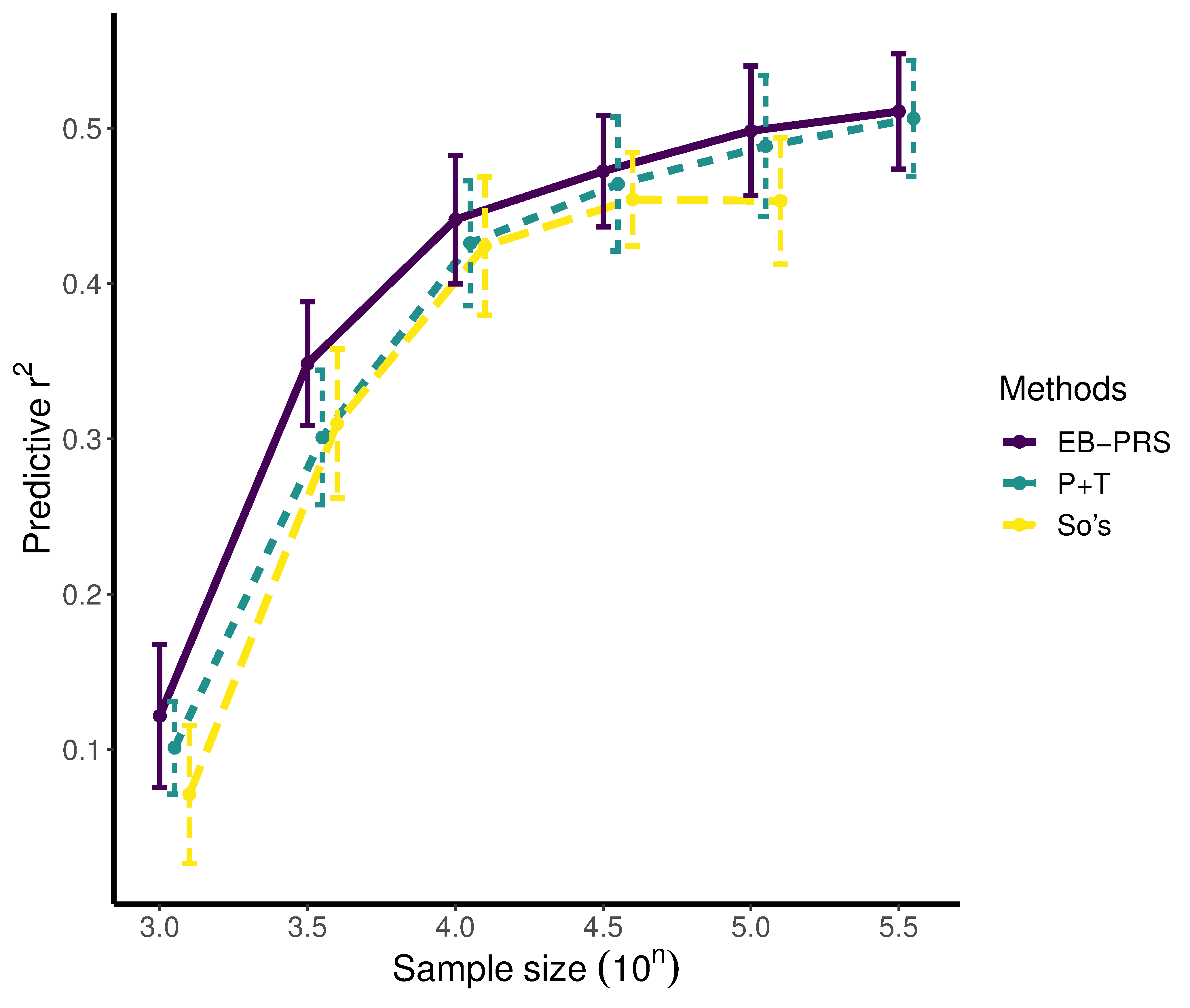

Supplement: S1 Fig — Here the control-to-case ratio is set to one. EB-PRS always outperformed the other methods. So et al.’s package will produce errors when the sample size is above 3e + 05. The error bar indicates the standard deviation of predictive r2 across 10 times simulations. (TIF) [file pcbi.1007565.s007.tif]

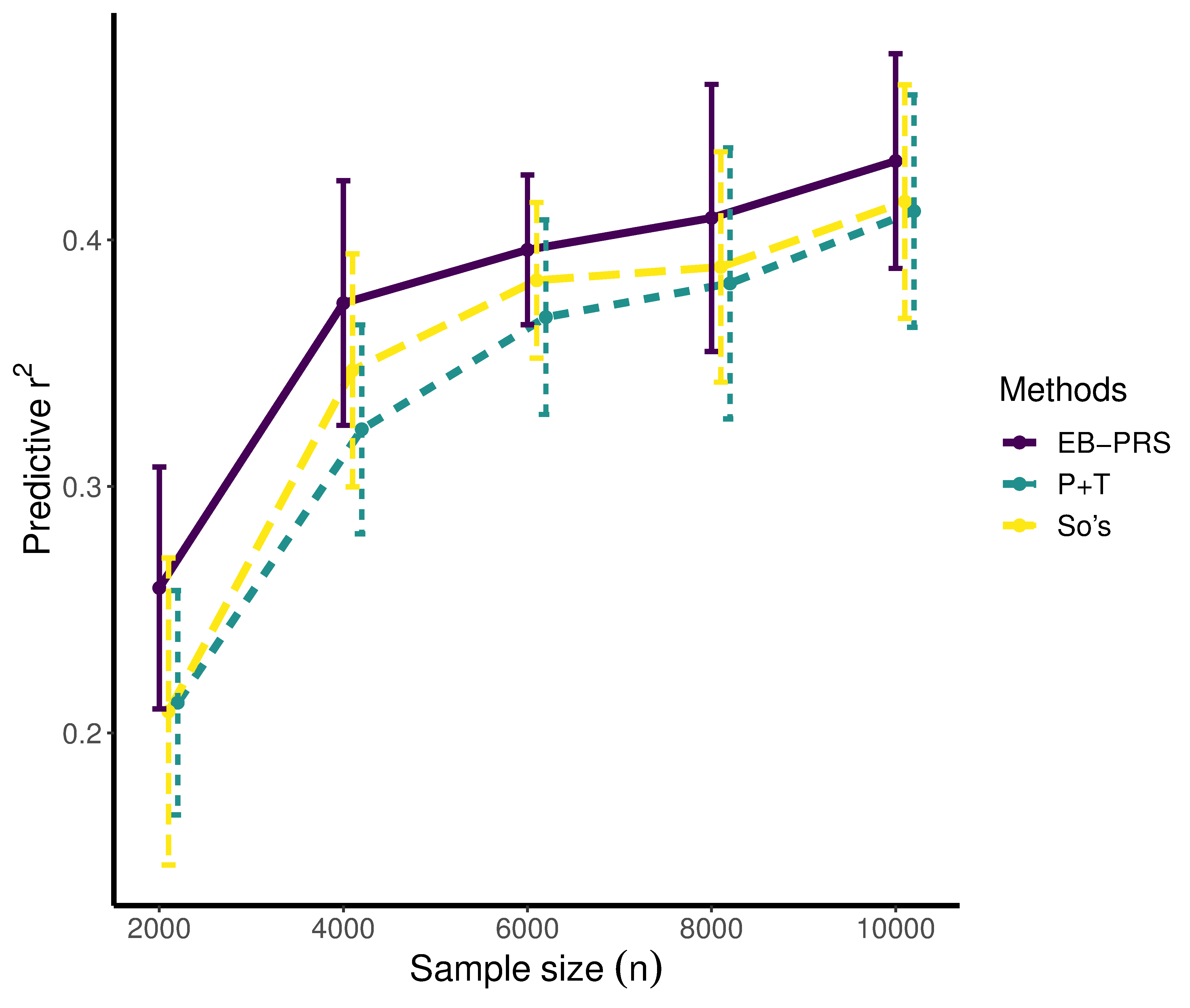

Supplement: S2 Fig — Here the control-to-case ratio is set to one. EB-PRS always outperformed the other methods. The error bar indicates the standard deviation of predictive r2 across 10 times simulations. (TIF) [file pcbi.1007565.s008.tif]

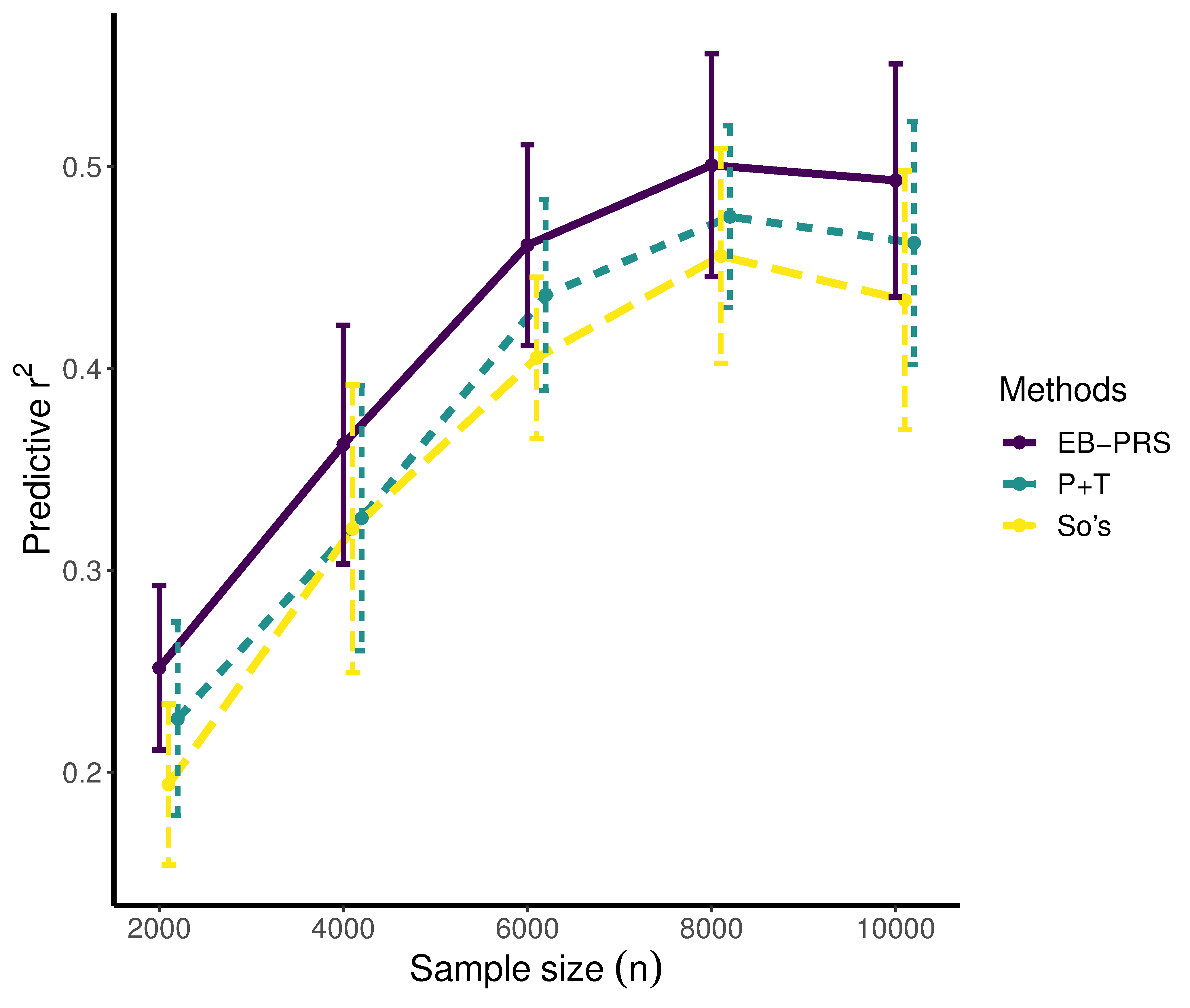

Supplement: S3 Fig — Here the control-to-case ratio is set to one. EB-PRS always outperformed the other methods. The error bar indicates the standard deviation of predictive r2 across 10 times simulations. (TIF) [file pcbi.1007565.s009.tif]

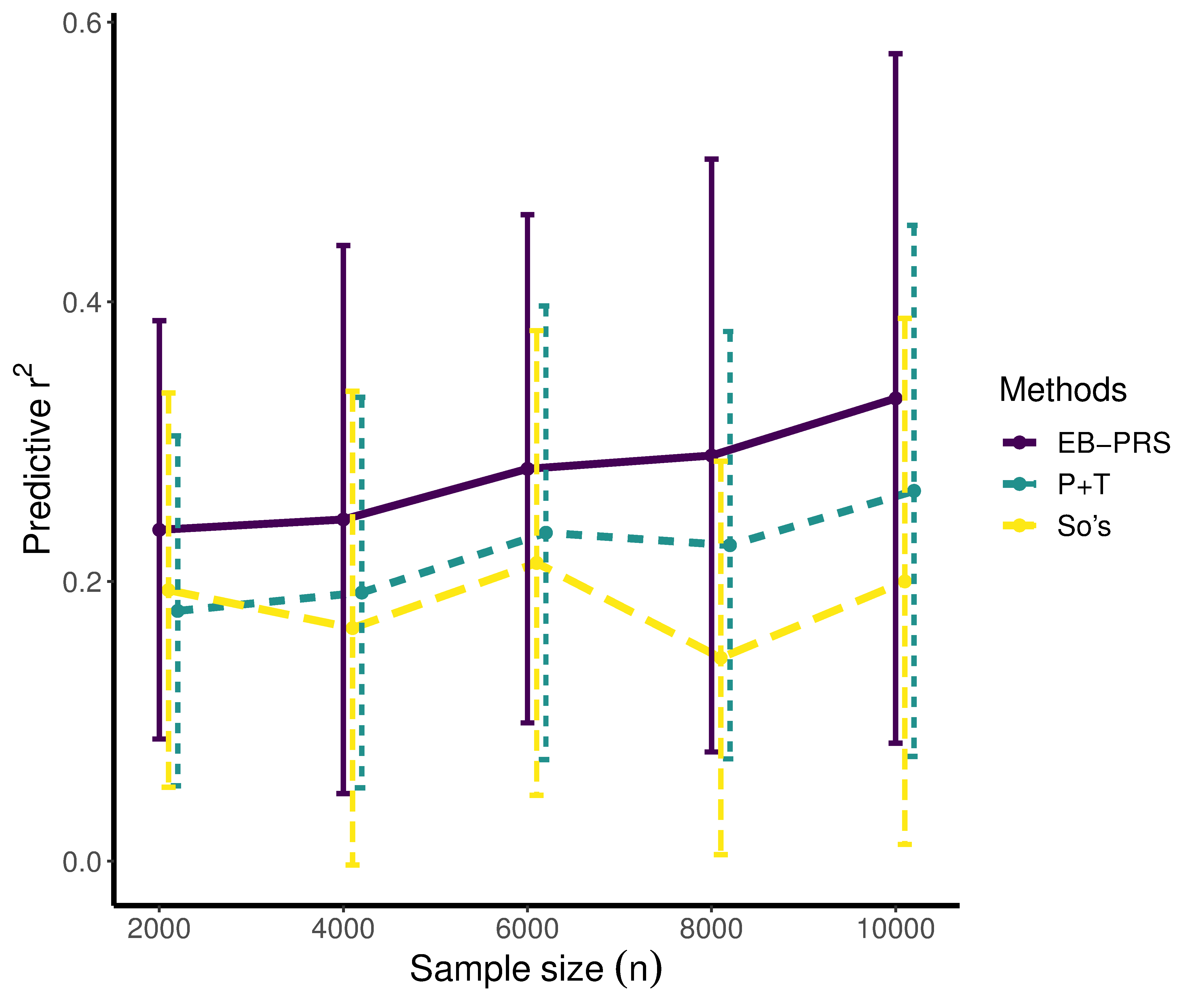

Supplement: S4 Fig — Here the control-to-case ratio is set to one. EB-PRS always outperformed the other methods. The error bar indicates the standard deviation of predictive r2 across 10 times simulations. (TIF) [file pcbi.1007565.s010.tif]
